# Prevalence of hypertension and related risk factors among children and adolescents at three separate visits: A large school-based study in China

**DOI:** 10.3389/fped.2022.976317

**Published:** 2022-09-23

**Authors:** Jia Hu, Ziyao Ding, Di Han, Bo Hai, Huiling Lv, Jieyun Yin, Hui Shen, Aihua Gu, Haibing Yang

**Affiliations:** ^1^State Key Laboratory of Reproductive Medicine, School of Public Health, Nanjing Medical University, Nanjing, China; ^2^Suzhou Institute of Advanced Study in Public Health, Gusu School, Nanjing Medical University, Suzhou, China; ^3^Suzhou Center for Disease Prevention and Control, Suzhou, China; ^4^Institute of Child and Adolescent Health, School of Public Health, Peking University, Beijing, China; ^5^Jiangsu Key Laboratory of Preventive and Translational Medicine for Geriatric Diseases, School of Public Health, Medical College of Soochow University, Suzhou, China

**Keywords:** adolescents, blood pressure, children, hypertension, risk factors, three visits

## Abstract

**Objective:**

We aimed to demonstrate characteristics of hypertension at three separate visits and its risk factors among children and adolescents based on a large school-based study in China.

**Materials and methods:**

Based on a large-scale ongoing monitoring program conducted in Suzhou, China, 59,679 children and adolescents aged 7–17 years from 60 public schools were enrolled during 2020 to 2021. Height, weight, and blood pressure (BP) were measured. Additional BP would be measured for hypertensive students at least 2 weeks later. Confirmed hypertension was defined as simultaneously BP meeting above or equal to 95th percentile for age, sex, and height at three separate visits. Odds ratio (ORs), and 95% CIs were calculated by logistic regression to identify risk factors for hypertension.

**Results:**

Prevalence of hypertension at three separate visits were 20.4, 6.3, and 3.1%, respectively. Prevalence of confirmed isolated systolic hypertension, isolated diastolic hypertension, and systolic and diastolic hypertension were 1.9, 0.3, and 0.9%, respectively. Hypertension prevalence for stages 1 and 2 were 2.6 and 0.6%. Different hypertension prevalence were found among various age and gender groups. Boys [OR, 1.137 (95% CI, 1.033–1.251)], high age [OR, 3.326 (95% CI, 2.950–3.751)], urban residents [OR, 1.579 (95% CI, 1.417–1.760)], high-socioeconomic status [OR, 1.156 (95% CI, 1.039–1.286)] and body mass index category including overweight [1.883 (95% CI, 1.665–2.129)], obesity [4.049 (95% CI, 3.625–4.523)], and thinness [OR, 0.457 (95% CI, 0.306–0.683)] were associated with confirmed hypertension.

**Conclusion:**

A single BP measurement would overestimate hypertension prevalence, about 3% Chinese children were hypertensive, early, and effective intervention around risk factors for hypertension should be taken.

## Introduction

Globally, hypertension is one of the important risk factors for cardiovascular diseases (CVDs) and related premature deaths (1). It is worth noting that individuals with high blood pressure (BP) in childhood tend to have high BP in adulthood (2, 3), the occurrence of hypertension in adolescence is closely related to future target organ injuries (such as coronary artery calcification, ventricular hypertrophy, and increased carotid intima-media thickness) (4, 5). Previous studies have indicated that earlier onset of HTN would bring longer treatment cycle, harder BP control, and worse prognosis (2, 6). Therefore, understanding the prevalence and risk factors of hypertension among children and adolescents has the important public health and clinical significance (7).

Blood pressure is affected by physical and psychological state, white coat, and other aspects, which is prone to false positive of hypertension, resulting in unnecessary medical treatment, family burden, and psychological panic. Standardized BP measurement is essential to accurately understand the status of hypertension in children and adolescents. The American Association of Pediatrics “Clinical Practice Guideline for Screening and Management of High Blood Pressure in Children and Adolescents” (AAP2017) (4) and 2018 Chinese health standard (CHS2018) “National Blood Pressure Reference for Chinese Han Children and Adolescents Aged 7–17 Years” (8) recommended that hypertension definition in children and adolescents should be based on at least three separate visits, with an interval of no less than 2 weeks. However, most epidemiological surveys about prevalence of hypertension have been conducted at only one occasion. At present, global prevalence of hypertension based on three separate visits among children and adolescents aged 6–19 years is 4.0% (9). However, limited studies based on three separate visits in China have been carried out. A study enrolled 44,396 urban children aged 6–17 from six major cities in China, and indicated that prevalence of hypertension was 4.0% (10). Another survey involving 7,786 adolescents aged 12–17 in two cities showed that prevalence of hypertension was 5.9% based on AAP2017 (11). We can find hypertension prevalence varied in different areas of China, considering China’s vast territory and large population, relevant research is still insufficient, especially in developed areas with a dense population.

Therefore, we conducted a large school-based study in Suzhou, a metropolis of the Yangtze River Delta in China, aiming to evaluate the prevalence of hypertension and hypertension subtype at three separate visits, and identify relevant risk factors.

## Materials and methods

### Study population

The current study was based on a large-scale ongoing school-based monitoring program (HPPCA) conducted in Suzhou, China. Detailed information about HPPCA has been published elsewhere (12). In short, HPPCA provided free annual health check-ups for all school-based students aged 6–17 years in Suzhou to assess the growth and development of children and adolescents. Physical examinations were conducted at general hospitals, disease prevention and control centers, or community healthcare facilities.

During 2020 to 2021, 60 public schools, including two primary schools, two junior high schools and two senior high schools from 10 counties/districts, were chosen using stratified random sampling method. All the students from the selected schools were invited to participate in the survey. Notably, participants with significant organ (i.e., heart, lung, liver, kidney) diseases and abnormal body changes, disabilities, and deformities were not included in this study. Finally, a total of 59,679 children and adolescents aged 7–17 years were included in this present study after excluding incomplete anthropometric information (*n* = 136).

All HPPCA work was carried out with the consent of participants and their parents. This study was approved by the Ethics Committee of Suzhou Center for Disease Prevention and Control.

### Body mass index measurements and definitions

All the physical examinations were carried out by professionally trained health workers using the same type of age-appropriate equipment and following the same procedures. Weight and height were measured with children in light clothing and without shoes. Height and weight were accurate to 0.1 cm and 0.1 kg, respectively. Body mass index (BMI) was calculated as weight (kg) divided by the square of the height (m) (12). Underweight, normal weight, overweight, and obesity were defined according to the latest Chinese Health standards of age- and sex-specific BMI cutoffs for children and adolescents, respectively (13, 14).

### Blood pressure measurements and definitions

Systolic BP (SBP) and diastolic BP (DBP) were measured using validated oscillometric devices (OMRON HBP-1300, HBP-1320, etc.) after each subject had rested for at least 15 min in a sitting position. An appropriate cuff was used to measure BP in children and adolescents. In total, three consecutive BP values were measured at 2-min intervals each time, and the average of the two closest BP readings was recorded.

Hypertension was defined as systolic BP (SBP) and/or diastolic BP (DBP) equal to or above the age-, sex-, and height-specific 95th percentile according to CHS2018 (8). Participants with hypertension at the first visit should be underwent a second BP measurement at least 2 weeks later; if BP was more than 95th percentile at the second visit, a third BP measurement should be taken following the same process. Confirmed hypertension was defined as simultaneously BP meeting above or equal to 95th percentile at three separate visits.

In order to distinguish hypertension phenotypes, isolated systolic hypertension (ISH) was defined as SBP ≥ P_95_ and DBP < P_95_, isolated diastolic hypertension (IDH) was defined as DBP ≥ P_95_ and SBP < P_95_, while systolic and diastolic hypertension (SDH) was defined as SBP ≥ P_95_ and DBP ≥ P_95_. Meanwhile, children with hypertension was be staged. Stage 1 is defined by BP between the 95th and 99th percentiles plus 5 mm Hg. Stage 2 is defined by BP ≥ 99th percentile plus 5 mm Hg.

### Statistical methods

Continuous variables were described as means ± SDs, while categorical variables were presented as numbers and percentages. Considering the differences in the age and sex distributions between our sample and the total population, we standardized the prevalence estimates by age and sex on the basis of 2020 Suzhou population census data.^1^ In order to compare with other researches, prevalence of hypertension were presented based on CHS2018 (8), AAP2017 (4), and CHL2018 (15), respectively. Estimate values (95% CIs) were presented.

In total, two sample Student’s *t*-tests (for continuous variables) or chi-square test (for categorical variables) were used to compare the differences across subgroups. Various socioeconomic status (SES) including relatively high SES (HSES) and relatively low SES (LSES) were distinguished based on GDP per capita from the statistics in Suzhou.^2^ Binary logistic regression was performed to identify related factors using hypertension at three visits as the dependent variable after adjustment for related covariates. All the statistical analyses were performed in R 3.2.2 software. The statistical significant level was defined as *P*-values less than 0.05 (2-sided).

## Results

### General characteristics of the study population

In total, 59,679 children and adolescents aged 7–17 years were included in our analysis at the first visit, mean age was 12.3 ± 3.0 years. Among them, 31,106 (52.1%) were boys, 28,750 (48.2%) were urban residents, and 31,261 (52.4%) were among LSES group. Mean values of SBP and DBP were 110.5 ± 13.4 and 68.7 ± 8.6 mm Hg, respectively. Prevalence of underweight, overweight, and obesity were 4.5, 18.1, and 15.8%, respectively (Table 1).

**TABLE 1:** Basic characteristics of all the participants.

Characteristics	All	Boys	Girls	*t*/χ^2^-value	*P*-value
*N*	59,679	31,106	28,573		
Age (year)	12.3 ± 3.0	12.3 ± 3.0	12.3 ± 3.0	−2.922	0.003
Height (cm)	153.6 ± 16.5	155.7 ± 18.0	151.2 ± 14.3	33.816	<0.001
Weight (kg)	48.6 ± 16.9	51.5 ± 18.7	45.5 ± 14.0	44.763	<0.001
BMI (kg/m^2^)	20.0 ± 4.0	20.5 ± 4.3	19.4 ± 3.6	35.038	<0.001
BMI status				2112.763	<0.001
Thinness	2,668 (4.5)	1,564 (5.0)	1,104 (3.9)		
Normal weight	36,765 (61.6)	16,499 (53.0)	20,266 (70.9)		
Overweight	10,797 (18.1)	6,666 (21.4)	4,131 (14.5)		
Obesity	9,449 (15.8)	6,377 (20.5)	3,072 (10.8)		
Region				2.370	0.124
Urban	28,750 (48.2)	15,079 (48.5)	13,671 (47.8)		
Rural	30,929 (51.8)	16,027 (51.5)	14,902 (52.2)		
SES				6.971	0.008
LSES	31,261 (52.4)	16,133 (51.9)	15,128 (52.9)		
HSES	28,418 (47.6)	14,973 (48.1)	13,445 (47.1)		
The first visit					
SBP (mmHg)	110.5 ± 13.4	113.0 ± 13.8	107.8 ± 12.3	48.259	<0.001
DBP (mmHg)	68.7 ± 8.6	68.6 ± 8.7	68.7 ± 8.4	−0.755	0.450
The second visit^a^					
SBP (mmHg)	115.6 ± 16.0	118.0 ± 16.8	112.8 ± 14.4	17.988	<0.001
DBP (mmHg)	70.9 ± 9.5	70.7 ± 9.6	71.2 ± 9.4	−2.494	0.013
The third visit^b^					
SBP (mmHg)	123.1 ± 14.5	126.1 ± 15.1	119.5 ± 13.0	14.138	<0.001
DBP (mmHg)	73.8 ± 9.2	73.7 ± 9.3	73.9 ± 9.1	−0.755	0.451

^*a*^*N* = 11,788; ^*b*^*N* = 3,707.

Compared with boys, girls had lower height, weight, and BMI (*P* < 0.001). There was no significant difference in residential region between boys and girls (*P* = 0.124). LSES group seemed to have higher portion of boys than HSES groups (*P* = 0.008). At all three visits, boys obviously had higher SBP than girls (*P* < 0.001), while girls had higher DBP than boys at second visit (*P* = 0.013), DBP did not show significant differences between boys and girls at first visit and third visit (*P* > 0.05).

### Prevalence of hypertension at three separate visits

**FIGURE 1:**
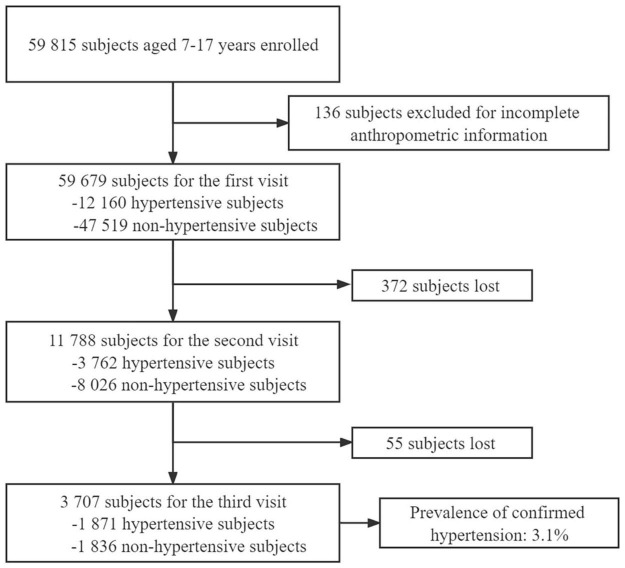
The flowchart of blood pressure measurements.

Based on the CHS2018, we identified 12,160 hypertensive subjects at the first visit, with a crude prevalence of 20.4% (95% CI, 20.1–20.7%), the crude prevalence of hypertension at the second visit was 6.3% (95% CI, 6.1–6.5%). After 3 separate visits, 1,871 subjects had high BP, the crude and standardized prevalence of confirmed hypertension was 3.1% (95% CI, 3.0–3.3%]), and 2.9% (95% CI, 2.8–3.1%), respectively (Figure 1 and Table 2). Similar decreased trends of hypertension prevalence with three visits were found in boys and girls (Supplementary Table 1).

**TABLE 2:** The crude and standardized prevalence of hypertension at three separate visits.

Characteristics	The first visit		The second visit		Confirmed hypertension	
	Crude (95% *CI*)	Standardized (95% *CI*)	Crude (95% *CI*)	Standardized (95% *CI*)	Crude (95% *CI*)	Standardized (95% *CI*)
Hypertension	20.4 (20.1, 20.7)	20.3 (20.0, 20.7)	6.3 (6.1, 6.5)	5.9 (5.7, 6.1)	3.1 (3.0, 3.3)	2.9 (2.8, 3.1)
Stage 1 hypertension	17.9 (17.6, 18.2)	17.9 (17.6, 18.3)	5.0 (4.8, 5.2)	4.7 (4.6, 4.9)	2.6 (2.4, 2.7)	2.4 (2.3, 2.6)
Stage 2 hypertension	2.5 (2.4, 2.6)	2.4 (2.3, 2.5)	1.3 (1.2, 1.4)	1.2 (1.1, 1.3)	0.6 (0.5, 0.6)	0.5 (0.5, 0.6)
ISH	10.3 (10.0, 10.5)	9.7 (9.5, 10.0)	3.4 (3.3, 3.6)	3.1 (3.0, 3.3)	1.9 (1.8, 2.0)	1.7 (1.6, 1.8)
IDH	6.3 (6.1, 6.4)	6.8 (6.5, 7.0)	1.1 (1.0, 1.2)	1.1 (1.0, 1.2)	0.3 (0.3, 0.4)	0.3 (0.3, 0.3)
SDH	3.9 (3.7, 4.0)	3.9 (3.7, 4.0)	1.8 (1.7, 2.0)	1.7 (1.6, 1.8)	0.9 (0.8, 1.0)	0.9 (0.8, 1.0)

The prevalence of crude hypertension was 5.8% (95% CI, 5.2–6.4%) using AAP2017 and 4.9% (95% CI, 4.3–5.5%) using CHL2018, both of which were significantly higher than the estimate (3.1%) based on the CHS2018 (*P* < 0.001). Similar results were found among boys and girls (Supplementary Table 2).

### Prevalence of hypertension phenotypes at three separate visits

Prevalence of hypertension phenotypes defined by CHS2018 were presented from the first visit to the third visit as in Table 2 and Supplementary Table 1. In total, the confirmed hypertension prevalence for stages 1 and 2 were 2.6 and 0.6%. While, the prevalence of confirmed ISH, IDH, and SDH were 1.9, 0.3, and 0.9%, respectively.

**FIGURE 2:**
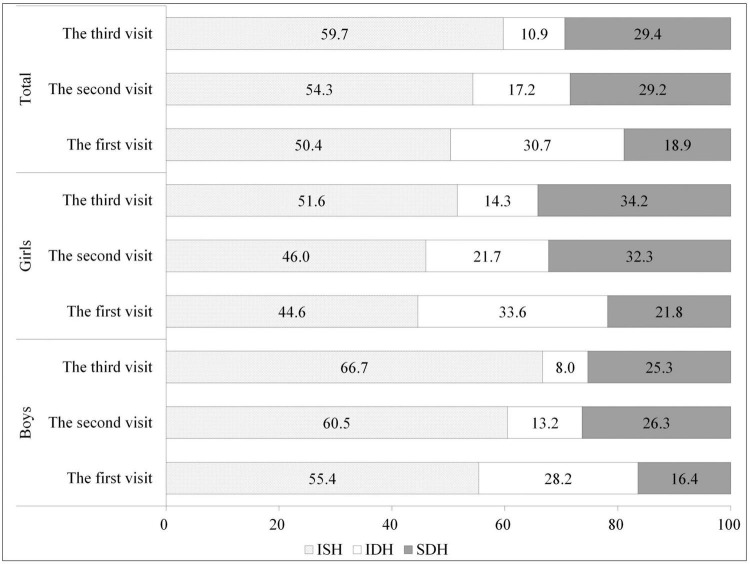
Proportions of hypertension phenotypes at three visits by gender group.

In total, the proportions of ISH slightly increased from visit 1 to visit 3 (50.4, 54.3, and 59.7%, respectively), and the proportions of IDH slightly decreased (30.7, 17.2, and 10.9% from visit 1 to visit 3, respectively), while the proportions of SDH increased from visit 1 to visit 2, and stabilized at visit 3 (18.9, 29.2, and 29.4%, respectively). Similar trends were found in both boys and girls (Figure 2). Proportions of hypertension phenotypes by age group and weight status at the third visit were presented (Supplementary Figure 1). Boys aged 12–17 years old and girls aged 7–11 years old had the highest proportion of ISH, overweight boys had the highest proportion of ISH, while normal weight girl had the highest proportion of ISH.

### Risk factors for hypertension at three separate visits

As shown in Table 3, high age, urban, HSES, thinness, overweight, and obesity were associated with hypertension at first visit. At the third visit, boys [OR, 1.137 (95% CI, 1.033–1.251)], high age [OR, 3.326 (95% CI, 2.950–3.751)], urban residents [OR, 1.579 (95% CI, 1.417–1.760)], HSES [OR, 1.156 (95% CI, 1.039–1.286)], and adiposity including overweight [OR, 1.883 (95% CI, 1.665–2.129)], and obesity [OR, 4.049 (95% CI, 3.625–4.523)] were associated with increased risk of confirmed hypertension. While those with thinness had a lower risk [OR, 0.457 (95% CI, 0.306–0.683)], when compared with normal weight subjects. We evaluated these risk factors across three separate visits; and found different risk levels at different visits. For example, the OR of obesity to hypertension increased from the first visit [2.374 (95% CI, 2.253–2.501)] to the third visit [4.049 (95% CI, 3.625–4.523)] (*P* < 0.001).

**TABLE 3:** Multivariate logistic regression analyses of the risk factors for hypertension at three separate visits.

Characteristics	The first visit		The second visit		Confirmed hypertension	
	*OR* (95% *CI*)	*P*-value	*OR* (95% *CI*)	*P*-value	*OR* (95% *CI*)	*P*-value
Gender						
Boys	Ref		Ref		Ref	
Girls	1.031 (0.990, 1.075)	0.144	1.085 (1.013, 1.162)	0.020	1.137 (1.033, 1.251)	0.008
**Age group**						
7–11	Ref		Ref		Ref	
12–17	1.114 (1.068, 1.162)	<0.001	2.354 (2.177, 2.546)*	<0.001	3.326 (2.950, 3.751)*^†^	<0.001
Region						
Urban	Ref		Ref		Ref	
Rural	1.325 (1.265, 1.387)	<0.001	1.434 (1.327, 1.549)	<0.001	1.579 (1.417, 1.760)*	<0.001
GDP						
Low	Ref		Ref		Ref	
High	1.187 (1.133, 1.243)	<0.001	1.074 (0.995, 1.160)*	0.067	1.156 (1.039, 1.286)	0.008
**BMI status**						
Thinness	0.662 (0.587, 0.747)	<0.001	0.615 (0.486, 0.778)	<0.001	0.457 (0.306, 0.683)*	<0.001
Normal weight	Ref		Ref		Ref	
Overweight	1.513 (1.436, 1.595)	<0.001	1.697 (1.555, 1.853)*	<0.001	1.883 (1.665, 2.129)*	<0.001
Obesity	2.374 (2.253, 2.501)	<0.001	3.322 (3.063, 3.603)*	<0.001	4.049 (3.625, 4.523)*^†^	<0.001

*Compared with the first visit, *P* < 0.05;

^†^Compared with the second visit, *P* < 0.05.

## Discussion

To our knowledge, this is the largest study to investigate hypertension prevalence after three separate visits and its risk factors among school-based children and adolescents in China. It provides BP status reference for developed areas in China. We also found that boys, high age, urban residents, HSES, and abnormal weight (thinness, overweight, and obesity) were associated with confirmed hypertension.

Most of the previous studies reporting the prevalence of hypertension among children and adolescents have been conducted at only one occasion. Based on the China Health and Nutrition Survey in 2015, prevalence of hypertension among children and adolescents aged 7–17 years was 19.2% defined by CHL2018 (16), and 12.8% defined by CHS2018 (17). These results were significantly higher than our results, which might be explained mainly by the number of BP visits. Substantial decreases in the prevalence of hypertension in children across different visits were reported by previous studies. A school-based survey performed in 7,832 Chinese children and adolescents showed that the prevalence of hypertension decreased substantially across three separate visits, with the prevalence of 17.2, 8.6, and 4.9%, respectively (18). In addition, a meta-analysis including 21 studies with 179,561 subjects aged 3–20 years showed that the prevalence of hypertension was 12.1, 5.6, and 2.7% across the three separate visits (19). In this study, prevalence of hypertension also decreased similarly across three visits. In sum, about 70–80% subjects with hypertension at the first visit returned to normal BP at the third visit. Overestimate of hypertension prevalence among children would result in psychological panic of parents and children, and unnecessary medical and family burden. These findings emphasized that BP should be measured on at least three different occasions to estimate the true prevalence of hypertension, which was also recommended by CHS2018, AAP2017, and CHL2018.

A survey involving 7,786 adolescents aged 12–17 years in two cities showed that the prevalence of hypertension was 5.9% based on AAP2017, and 8.4% based on CHL2018 (11), both of which were higher than results in this study using same references. Other studies conducted in China such as Jinan (4.9%) (18) and Chongqing (6.0%) (20) also had higher prevalence of hypertension. Recently, a systematic review and meta-analysis included 25 studies of 341,281 participants reported the prevalence of hypertension among Chinese children was 9.8% (95% CI: 7.9–11.9%) (21). Another meta- analysis included 39 articles of 796,512 subjects indicated hypertension prevalence was 9.1% (95% CI: 7.4–10.7%) (22). The great discrepancy with our study might be the uncertain number of BP visits and the BP references in the review. A review reported that global prevalence of hypertension based on three separate visits from 47 researches of 186,630 children and adolescents aged 6–19 years was 4.00% (95% CI, 3.29–4.78%) (9), another previous review report included 21 studies with 179,561 children aged 3–20 years was 2.7% (95% CI = 2.1–3.3%) (19). Our result lay between these two results (9, 19).

Overweight/obesity have been found to increase the risk of hypertension at three visits. With the increases of BP follow-up times, the correlations between overweight and obesity with hypertension increased compared with the normal children. The Chinese National Survey on Students’ Constitution and Health (1995–2014) showed that the attributable risk ratio of overweight and obesity with hypertension was 19.2% in 2014 (23). The obesity-induced adolescent hypertension may be mediated partly by the activation of the sympathetic nervous system, which included insulin resistance and secretion of leptin. It highlights that keeping a healthy weight can serve as an important intervention to control BP level in children and adolescents. Boys had higher prevalence of elevated BP than girls in our study, higher prevalence of overweight and obesity among boys may explain this phenomenon to some extent (24). Meanwhile, adolescents aged 12–17 years had higher risk of hypertension than children aged 7–11 years, and hypertension prevalence of children aged 7–11 years decreased significantly greater at the second and third visit than adolescents aged 12–17 years. Possible explanations for the age differences included intense hormonal changes and elevated insulin resistance during pubertal period. It suggested that repeated blood pressure measurements had a greater significance for the low age group. Relative higher hypertension prevalence in adolescents determined that hypertension detection of adolescents of the high age group should be taken as the key management population, which is consistent with previous study (10, 18).

Considering regional and ethnic differences in BP, in order to compare with other researches, we also applied AAP2017 and CHL2018 to evaluate hypertension prevalence. Compared with the results using CHS2018, the prevalence of hypertension across three visits were higher applying the AAP2017, and results of CHL2018 were the highest. Our findings were consistent with the results with relevant studies based on a single BP visit (25, 26). However, relevant comparisons using CHS2018 were limited. A study among the Chinese children and adolescents evaluated hypertension prevalence across three visits, and showed that results of CHL2018 were higher than those using AAP2017 (11). The divergence in height distributions may explain the difference in hypertension prevalence applying various references. Great height differences for both males and females at the same percentile among various references existed, and the gap increased in late puberty, especially. These differences would bring different BP values of the same age, sex, and height percentile (25). One point needs to be noted, a statistical definition is still the principal method for childhood hypertension evaluation. In the future, longitudinal studies should be conducted to explore the clinical effects of different BP levels, which would be useful for accurate assessment of BP (27).

Previous studies indicated that young adults with ISH, IDH, and SDH were all positively associated with higher risks of CVD than non-hypertensive persons, with SDH being the highest, and IDH higher or close to ISH (28, 29). Nevertheless, impact of ISH, IDH, and SDH during childhood on future CVD are still limited, identification of children with ISH, IDH, and SDH may improve to identify risk level. ISH usually reflects essential hypertension (30). In the present study, it was the most frequent phenotype, its proportion increases with times of BP measurements. Interestingly, there were substantial differences in the proportions of hypertension subtypes between boys and girls, such as 66.7% of ISH in boys and 51.6% of ISH in girls at third visit (30). We spectated that puberty and hormones may play a very important role on hypertension development. Previous study indicated estrogen was essential for the enhancement of endothelium-dependent vasodilation and regulation of smooth muscle cells.

Our study had several strengths. To our knowledge, this is the first study to report hypertension prevalence across three separate visits in eastern China using various references. Besides, the second and third visits were completed by the school’s healthcare personnel, keeping this school-based study with high-participation rate to minimize selection bias (second visit: 96.9%, third visit: 98.5%). In addition, BP measurements with favorable environment by familiar school personnel reduced white-coat hypertension and masked hypertension.

However, limitations of the present study should be noted. First, only children whose BP at least 95th percentile were followed to have the subsequent BP measurements, which would result in underestimated prevalence of hypertension. Second, since students were from only 1 developed region, the generalizability of the present findings might be limited to represent developed regions in China, and this needs to be confirmed. Third, we did not collect information about lifestyle factors (eating behavior, salt intake, and physical activity), mental health, and environmental exposures. Finally, a cross-sectional study design would limit us to identify causal associations of hypertension with risk factors, and health effects of hypertension should be further evaluated.

## Data availability statement

The raw data supporting the conclusions of this article will be made available by the authors, without undue reservation.

## Ethics statement

The studies involving human participants were reviewed and approved by the Ethics Committee of Suzhou Center for Disease Prevention and Control. Written informed consent to participate in this study was provided by the participants’ legal guardian/next of kin.

## Author contributions

HY and AG contributed to the design and concept of the manuscript. JH was responsible for the analysis, interpretation of data, and manuscript drafting. DH, ZD, and BH organized the database. HL performed the statistical analysis. HS and JY were responsible for the critical revision of the manuscript for intellectual content. All authors wrote this manuscript and gave final approval of the submitted and published versions.

## Footnotes

1
http://www.stats.gov.cn/tjsj/pcsj/rkpc/6rp/indexch.htm

2
http://tjj.suzhou.gov.cn/
