# Supplementary material for: Prevalence of hypertension and related risk factors among children and adolescents at three separate visits: A large school-based study in China
Source: Front Pediatr. 2022 Sep 23;10:976317. doi: 10.3389/fped.2022.976317 (PMC9537608; doi:10.3389/fped.2022.976317)
Supplement: Supplementary file 1 [file Data_Sheet_1.docx]

Supplementary Material

Table 1s The crude and standardized prevalence of hypertension separated by gender at three separate visits

| Gender | Characteristics | The first visit | |  | The second visit | |  | Confirmed hypertension | |
| --- | --- | --- | --- | --- | --- | --- | --- | --- | --- |
|  |  | Crude (95% *CI)* | Standardized (95% *CI*) |  | Crude (95% *CI)* | Standardized (95% *CI*) |  | Crude (95% *CI)* | Standardized (95% *CI*) |
| Boys | Hypertension | 21.1(20.6, 21.5) | 21.1(20.5, 21.6) |  | 6.5(6.3, 6.8) | 6.1(5.6, 6.4) |  | 3.2(3.0, 3.4) | 2.9(2.7, 3.1) |
|  | Stage 1 hypertension | 18.3(17.9, 18.8) | 18.5(18.0, 19.0) |  | 5.2(4.9, 5.4) | 5.0(4.7, 5.2) |  | 2.6(2.4, 2.8) | 2.5(2.3, 2.7) |
|  | Stage 2 hypertension | 2.7(2.5, 2.9) | 2.6(2.4, 2.8) |  | 1.4(1.2, 1.5) | 1.3(1.1, 1.4) |  | 0.7(0.6,0.8) | 0.6(0.5, 0.7) |
|  | ISH | 11.7(11.3, 12.0) | 11.0(10.6, 11.4) |  | 4.0(3.7, 4.2) | 3.6(3.4, 3.9) |  | 2.2(2.0, 2.3) | 2.0(1.8, 2.1) |
|  | IDH | 5.9(5.7, 6.2) | 6.5(6.2, 6.9) |  | 0.9(0.8, 1.0) | 0.9(0.8, 1.1) |  | 0.3(0.2, 0.3) | 0.3(0.2, 0.4) |
|  | SDH | 3.5(3.3, 3.7) | 3.5(3.3, 3.8) |  | 1.7(1.6, 1.9) | 1.6(1.5, 1.8) |  | 0.8(0.7, 0.9) | 0.8(0.7, 0.9) |
| Girls | Hypertension | 19.6(19.2, 20.1) | 19.6(19.0,20.1) |  | 6.0(5.8, 6.3) | 5.6(5.3, 5.8) |  | 3.0(2.8, 3.2) | 2.7(2.5, 2.9) |
|  | Stage 1 hypertension | 17.4(16.9,17.8) | 17.4(16.9,17.9) |  | 4.9(4.6, 5.1) | 4.5(4.3, 4.8) |  | 2.6(2.4, 2.7) | 2.4(2.2, 2.6) |
|  | Stage 2 hypertension | 2.3(2.1,2.4) | 2.2(2.0,2.4) |  | 1.2(1.1, 1.3) | 1.1(1.0, 1.2) |  | 0.5(0.4, 0.5) | 0.4(0.4, 0.5) |
|  | ISH | 8.8(8.4, 9.1) | 8.4(8.0,8.7) |  | 2.8(2.6, 3.0) | 2.6(2.4, 2.8) |  | 1.6(1.4, 1.7) | 1.5(1.3, 1.6) |
|  | IDH | 6.6(6.3, 6.9) | 7.0(6.6,7.3) |  | 1.3(1.2, 1.4) | 1.2(1.1, 1.4) |  | 0.4(0.4, 0.5) | 0.4(0.3, 0.5) |
|  | SDH | 4.3(4.0, 4.5) | 4.2(4.0,4.5) |  | 1.9(1.8, 2.1) | 1.8(1.6, 1.9) |  | 1.0(0.9, 1.1) | 1.0(0.9, 1.1) |

Table 2s Prevalence of hypertension by three commonly used references at three separate visits [prevalence (95% CI)]

|  | The first visit | The second visit | Confirmed hypertension |
| --- | --- | --- | --- |
| Total |  |  |  |
| CHS2018 | 20.4(20.1, 20.7) | 6.3(6.1, 6.5) | 3.1(2.6, 3.7) |
| CHL2018 | 26.8(26.4, 27.1)^a^ | 10.0(9.5, 10.5) ^a^ | 5.8(5.2, 6.4) ^a^ |
| AAP2017 | 25.2(24.9, 25.6)^a, b^ | 8.9(8.4, 9.3) ^a, b^ | 4.9(4.3, 5.5) ^a, b^ |
| Boy |  |  |  |
| CHS2018 | 21.1(20.6, 21.5) | 6.5(6.3, 6.8) | 3.2(3.0, 3.4) |
| CHL2018 | 27.6(27.1, 28.1) ^a^ | 10.3(9.7, 10.9) ^a^ | 6.1(5.3, 6.9) ^a^ |
| AAP2017 | 27.6(27.1, 28.1) ^a^ | 10.1(9.5, 10.7) ^a^ | 5.7(4.9, 6.5) ^a^ |
| Girl |  |  |  |
| CHS2018 | 19.6(19.2, 20.1) | 6.0(5.8, 6.3) | 3.0(2.8, 3.2) |
| CHL2018 | 25.8(25.3, 26.3) ^a^ | 9.7(9.0, 10.3) ^a^ | 5.5(4.7, 6.4) ^a^ |
| AAP2017 | 22.7(22.2, 23.2) ^a, b^ | 7.6(7.0, 8.3) ^a, b^ | 4.1(3.3, 4.9) ^b^ |

^a^Compared to CHS2018, *P*<0.05, ^b^ Compared to CHL2018, *P*<0.05.


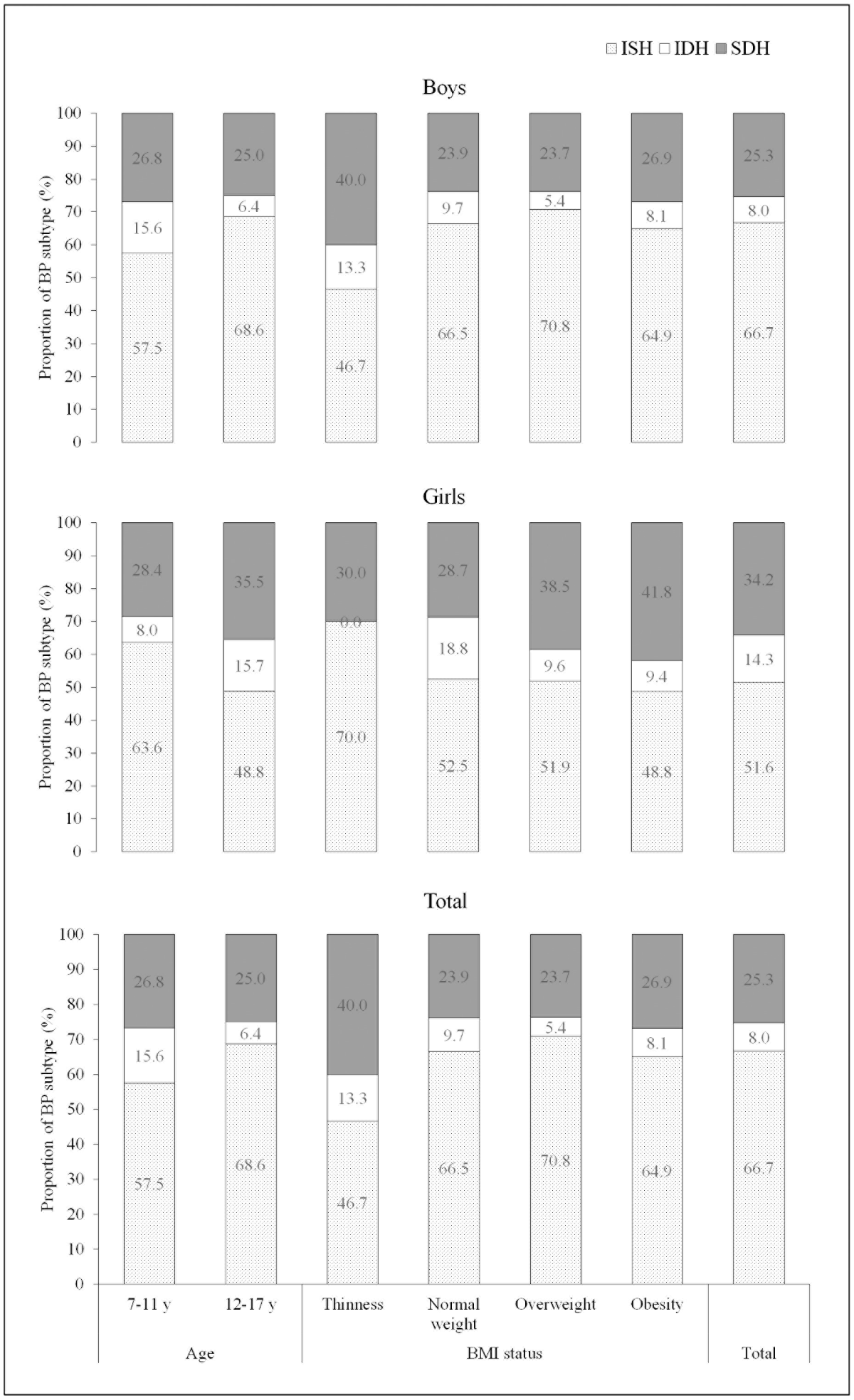


Figure 1S Proportions of hypertension phenotypes at the third visit by age and weight status among different gender groups.
